# Epidemiology of hospitalized pediatric bacterial sepsis in Brazil: a trend analysis from 1992 to 2006

**DOI:** 10.1186/cc10170

**Published:** 2011-06-22

**Authors:** CMF Mangia, N Kissoon, OA Branchini, MC Andrade, BI Kopelman, J Carcillo

**Affiliations:** 1 Universidade Federal de São Paulo, São Paulo - SP, Brazil

## Objective

To determine the epidemiology, costs and outcome of hospitalized pediatric sepsis in Brazil (1992 to 2006) and to compare mortality caused by sepsis with that caused by other major childhood diseases.

## Methods

We performed a population-based cohort study using a government database of all hospitals affiliated with the Brazilian health system. We studied all hospitalizations in children from 28 days through 19 years with diagnosis of bacterial sepsis defined by the criteria of the International Classification of Diseases.

## Results

From 1992 through 2006, the pediatric hospital mortality rate was 1.23%. There were 556,073 pediatric admissions with bacterial sepsis, with a mean mortality rate of 19.9%. The incidence of sepsis decreased 64% from 1992 to 2006 (*P *< 0.001); however, the mortality rate remained unchanged (from 1992 to 1996, 20.5%; and from 2002 to 2006, 19.7%). The sepsis hospital mortality rate was substantially higher than pneumonia (0.5%), HIV (3.3%), diarrhea (0.3%), undernutrition (2.3%), malaria (0.2%) and measles (0.7%). The Human Development Index and mortality rates by region were: North region 0.76 and 21.7%; Northeast region 0.72 and 27.1%; Central-West region 0.81 and 23.5%; South region 0.83 and 12.2%; and Southeast region 0.82 and 14.8%, respectively.

## Conclusion

Sepsis remains an important health problem in children in Brazil. The institution of universal primary care programs has been associated with substantially reduced sepsis incidence and therefore deaths; however, hospital mortality rates in children with sepsis remain unchanged. Implementation of additional health initiatives to reduce sepsis mortality in hospitalized patients could have great impact on childhood mortality rates in Brazil.

